# Supplementary material for: Phenotypic Spectrum of NFIA Haploinsufficiency: Two Additional Cases and Review of the Literature
Source: Genes (Basel). 2022 Nov 30;13(12):2249. doi: 10.3390/genes13122249 (PMC9777632; doi:10.3390/genes13122249)
Supplement: Supplementary file 1 [file genes-13-02249-s001.zip › genes-1961624-supplementary.pdf]

Supplementary Tables

Table S1. Other defects described.

	OTHER DEFECTS
P3	-hypothyroidism secondary to partial thyroidectomy, (the indication for partial thyroidectomy was not clear from the patient's history);<br>-late menarche, and delayed development of secondary sexual characteristics
P14	pulmonic stenosis
P16	polycystic ovarian syndrome
P17	diabetes, pseudopapilledema
P18	fibromyalgia, hyperthyroidism
P20	Decreased circulating antibody level
P21	Recurrent otitis media
P25	After birth, tachycardia

Additional defects of the selected patients.

Table S2. Excluded patients. Excluded patients were characterized by other genetic alterations along with to those involving *NFIA*, by an absent or scanty phenotype, and by large deletions harboring *NFIA* (>1.5 MB) and other genes. For each patient the corresponding reference (literature and databases), the genetic alterations and the extent of each alteration are reported. The intragenic deletions/duplications of *NFIA* are referred to *NFIA*-207 transcript (ENST00000403491.8; RefSeq ID NM_001134673).

	NFIA **+ ADDITIONAL GENETIC VARIANTS**	
Lu et al., 2007 [5] (DGAP089)	t(1;2) (p31.3;q22.1), breakpoint in 1p31.3 spanning intron 7 of *NFIA*	-2 Mb deletion in 2q14.3q21<br>-breakpoint in 1q22.1 spanning no coding sequence
Lu et al., 2007 [5] (DGAP104)	t(1;20) (p31.3;q13.31) breakpoint in 1p31.3 spanning intron 2 of *NFIA*	breakpoint in 20q13.3 1spanning of *C20orf32*
Coci et al., 2016 [26]	inv(1)(p36.31p31.3) breakpoint in 1p31.3 with deletion of *NFIA* exons 3-4	breakpoint in 1p36.31p31 with deletion *CAMTA*
285169 Decipher	419 Kb duplication in 1p31.3 (*NFIA* exons 3-11)	2.07 Mb deletion in 15q13.2q13.3
413383Decipher	383 Kb duplication in 1p31.3 (*NFIA* exons 3-11)	1.11 Mb deletion in 19p13.3
480090Decipher	473 Kb duplication in 1p31.3 (*NFIA* exons 3-11)	333 Kb duplication in 11q23.2 harboring *NCAM1*
Ogura et al., 2022 [x][27]	*NFIA* (p.Thr395Met; NM_001134673.4)	[46,XY,t(1;5)(p31.1;q13.3)t(2;6)(q24.2;q23.1)]
Revah-Politi et al., 2017	*NFIA* (p.Arg69Ter; NM_001145512.1)	*PTEN* (p.Pro96Ala; NM_000314.4)

[23] (case 4)		
263798Decipher	*NFIA* (p.Arg160Ter; ENST00000371189.8)	*SETD2* (p.Asp1713Gly; ENST00000409792.4)
305440 Decipher	*NFIA* (p.Trp26Ter; ENST00000371189.8)	-*MYH9* (p.Lys1248Glu; ENST00000216181.11)<br>-*COG4* (p.Arg502Trp; ENST00000323786.10)<br>-*COL4A3* (p.Gly118Arg; ENST00000396578.8)
Wongkittichote et al., 2022 [X][28]	*NFIA* (c.819-1G>A; IVS5-1G>A; NM_001134673.4)	-*SCN2A* (p. Met640Thr; NM_021007.2)
ABSENT OR SCANTY PHENOTYPE		
438681Decipher	410 Kb duplication in 1p31.3 (*NFIA* exons 3-11)	no phenotype
438724 Decipher	63 Kb deletion in 1p31.3 (*NFIA* exon 2)	no phenotype
480105 Decipher	418 Kbduplication in 1p31.3 (*NFIA* exons 3-11)	no phenotype
260253 Decipher	116 Kb deletion in 1p31.3 (*NFIA* exons 3-10)	no phenotype
274057 Decipher	89,75 Kb duplication in 1p31.3 (*NFIA* exons 10-11)	phenotype scanty (global developmental delay)
363965 Decipher	23 Kb deletion in 1p31.3 (*NFIA*intron 3) (CNV likely benign)	phenotype scanty (micropenis, mild global developmental delay)
342059 Decipher	3Kb deletion in 1p31.3 (*NFIA*intron 3) (CNV likely benign)	phenotype scanty (global developmental delay)
Iossifov et al.,2012 [29]	*NFIA* (p.Arg83Ter; ENST00000371189.8)	phenotype scanty (autistic spectrum)
LARGE DELETIONS INCLUDING *NFIA*		
Campbell at al.,2022 [10]; Lu et al., 2007 [5] (DGAP205–1) (DGAP205-1s)	12 Mb deletion in 1p31.2p32.3	
Lu et al., 2007 [5] (DGAP174)	-2.2 Mb deletion in1 p31.3p32.1<br>-t(1;3)(p31.1;q25.1)breakpoint in 1p31.1 spanning NEGR1 gene	
Koehler et al., 2010 [11]	4.93 Mb deletion in 1p31.3p32.2	
Chen et al., 2011 [12]	22,2 Mb deletion in 1p32.3p31.1	
Ji, Salamon, & Quintero-Rivera, 2014 [13]	22,88 Mb deletion in 1p31.1p32.2	
Labonne et al., 2016 [14]	9,45 Mb deletion 1p31.3p32.2	
Prontera et al., 2017 [15]	8,5 Mb deletion in 1p32.2p31.3	
353831 Decipher	11,67 Mb deletion in 1p32.1p31.3	
251391 Decipher	11,20 Mb deletion in 1p32.2p31.1	
276512 Decipher	8,83 Mb deletion 1p31.3-1p32.1	
282222 Decipher	7,99 Mb deletion 1p32.3p31.3	

317160 Decipher	7,21 Mb deletion 1p321.2p31.3
264827 Decipher	5,43 Mb deletion 1p31.3
261605 Decipher	4,98 Mb deletion 1p32.1p31.3
401017 Decipher	4,78 Mb deletion 1p32.2p31.3
379364 Decipher	15,12 Mb deletion1p32.2p31.1
2714 Decipher	13,77 Mb deletion 1p32.2p31.1
322751 Decipher	7,21 Mb deletion 1p32.2p31.3
4638 Decipher	1,54 Mb deletion 1p32.1p31.3
438670 Decipher	6,81 Mb deletion 1p32.1p31.3
285848 Decipher	14,14 Mb deletion 1p32.1p31.3

Table S3. Comparison between *NFIA*-207 and *NFIA*-205 transcripts.

***NFIA* *207* ex**	***NFIA205* ex**	**GRCh37/hg19**	**aa sequence**	**patients**	***NFIA-207* variants**	***NFIA-205* variants**
	1	61,547,534_61,547,719	MQMCRPASSSVLYVPTRWPGGCGATWQSCPSPPPR			
	2	61,548,433_61,548,490	RTRIPQRPAVMYSPLCLT*Q*	P16		p.Gln54ProfsTer49
				P21		c.163-2A>G SpliceAI: ΔS acceptor loss
1		61,548,233_61,548,490	MYSPLCLT*Q*	P16	p.Gln9ProfsTer49	
				P21	c.28-2A>G SpliceAI: ΔS acceptor loss	
2	3	61,553,821_61,554,352	DEFHPFIEALLPHVRAFAYTWFNLQARKRKYFKKHEKRMSKEEERAVKDELLSEKPEVKQKWASRLLAKLRKDIRPEYREDFVLTVTGKKPPCCVLSNPDQKGKMRRIDCLRQADKVWRLDLVMVILFKGIPLESTDGERLVKSPQCSNPGLCVQPHHIGVSVKELDLYLAYFVHAAD	P17-18	p. Arg24Ter	p. Arg69Ter
				P22	p.Arg38Ter	p.Arg83Ter
				P19	p. Arg74Ter	p. Arg119Ter
				P20	p.Leu75Pro	p.Leu120Pro
				P24	p.His167Arg	p.His212Arg
3	4	61,743,192_61,743,257	SSQSESPSQPSDADIKDQPEN*G*	P25-P26	p.Lys125Glu	p.Lys170Glu
4	5	61,798,184_61,798,258	HLGFQDSFVTSGVFSVTELVRVSQ*T*			
5	6	61,818,122_61,818,239	PIAAGTGPNFSLSDLESSSYYSMSPGAMRRSLPST			

			SSTS			
6	7	61,824,819_61,824,946	STKRLKSVEDEMDSPGEEPFYTGQGRSPGSGSQSS GWHEVEPG			
7	8	61,848,909_61,849,037	MPSPTTLKKSEKSGFSSPSPSQTSSLGTAFTQHH**R** PVITGPRA	P23	p.Arg351Ter	p.Arg396Ter
8	9	61,869,776_61,869,954	SPHAT**P**STLHFPTSPIIQQPGPYFSHPAIRYHPQE TLKEFVQLVCPDAGQQAGQVGFLN	P15	p.Pro365-Hisfs*32	p.Pro410 HisfsTer32
9	10	61,872,234_61,872,399	PNGSSQGKVHNPFLPTPMLPPPPPPPMARPVPLPV PDTKPPTTSTEGGAASPTSPT			
10	11	61,892,137_61,892,228	YSTPSTSPANRFVSVGPRDPSFVNIPQQTQ			
11	12	61,920,975_61,928,460	SWYLG			

These two transcripts differ in the 5' region. For each isoform is reported the exon number, the coding sequence position (GRCh37/hg19),along with the corresponding amino acid sequence. The variants of the patients are described according to *NFIA*-207 and *NFIA*-205. In the amino acid sequence (aa sequence) column the conserved domains [NfI_DNAbd_pre-N/pfam10524 (orange), MH1/cl00055 (green), CTF_NFI/pfam00859 (purple)]are also reported;the residues involved in the variations are highlighted, those overlap splice site are in italic. aa: amino acid; ex: exon; P: patient. *NFIA*-207 (ENST00000403491.8; RefSeq ID NM_001134673) (red); *NFIA*-205 (ENST00000403491.8; RefSeq ID NM_001145512.2)(blue).

Table S4. Neurodevelopmental details.

	DD/ID
P4	(MILD) The patient presented at 3 years with gross motor delay and delay of fine motor skills<br>Griffith's Developmental Assessment at that time was suggestive of poor locomotor and social skills and poor hand/eye coordination.<br>Her practical reasoning and speech skills were at a normal level; however, she had difficulties with articulation.
P6	(MILD) Developmental milestones:<br>age at walking independently 18 months,<br>age at first word 12 months,<br>and age at completing sentences 4 years.
P11	(MILD) Developmental milestones:<br>age at walking independently 18 months,<br>age at first word 18 months,<br>and age at completing sentences 3 years.
P15	(MILD) Developmental milestones:<br>age at holding his head up 4 months and<br>age at walking without support at 1 year and 3 months.<br>age at speaking meaningful words at 2 years and 1 month,<br>His intelligence quotient at 4 years measured by the Tanaka–Binet Intelligence Scale was 75 (in the borderline range)

P16	(NORMAL) Although she started speaking before 12 months of age,<br>she did not walk until about 17 months of age.<br>Neuropsychologic tests at 14 years of age showed her full-scale IQ (FSIQ) to be 97 (normal 94–102),<br>with a Verbal Comprehension (VCI) score in the high average range.<br>Her Perceptual Reasoning (PRI) and WorkingMemory (WMI) were both in the average range
P17	(SEVERE) She was noted to have speech delay at 24 months (at which time she spoke less than 20 words and could not form two-to-three word sentences).<br>At the age of 3 years, she was unable to walk up stairs or climb.
P19	(MODERATE/NORMAL) At the age of 3 month, he could not hold up his head; he was able to hold his head up at 6 months, sit alone at 10 months, and hold things to stand at 1 year old. He can call mom and dad with conscious at 1 year old.<br>At the age of 3 month, DDST (Denver DevelopmentalScreening Test) showed lower DQ (development quotient) (60, normal range >85), and lower MI (mental development index) (70, normal range >85).<br>At the age of 6 months, DDST showed a DQ of 83, and an MI of 89.
P25	(SEVERE) She started to walk at the age of 3 years.<br>She also stated to speak her words at the age of 3 years and spoke only few words at the age of 6 years.<br>Her developmental quotient as assessed using the WISC-IV test was 23
P26	(MILD) He gained head control and rolling over at 8 months of age,<br>sat without support at 11 months of age, and slithering at 1 year of age.<br>He was able to stand with support at 1 year of age

ID: intellectual disability; DD: developmental delay.
